# First person – Abdulsalam Isiaku

**DOI:** 10.1242/dmm.049180

**Published:** 2021-07-23

## Abstract

First Person is a series of interviews with the first authors of a selection of papers published in Disease Models & Mechanisms, helping early-career researchers promote themselves alongside their papers. Abdulsalam Isiaku is first author on ‘ Transient, flexible gene editing in zebrafish neutrophils and macrophages for determination of cell-autonomous functions’, published in DMM. Abdulsalam is a PhD candidate/graduate student in the lab of Prof. Graham Lieschke at Monash University, Clayton, Australia, investigating the role of phagocytes in inflammatory and infectious diseases.


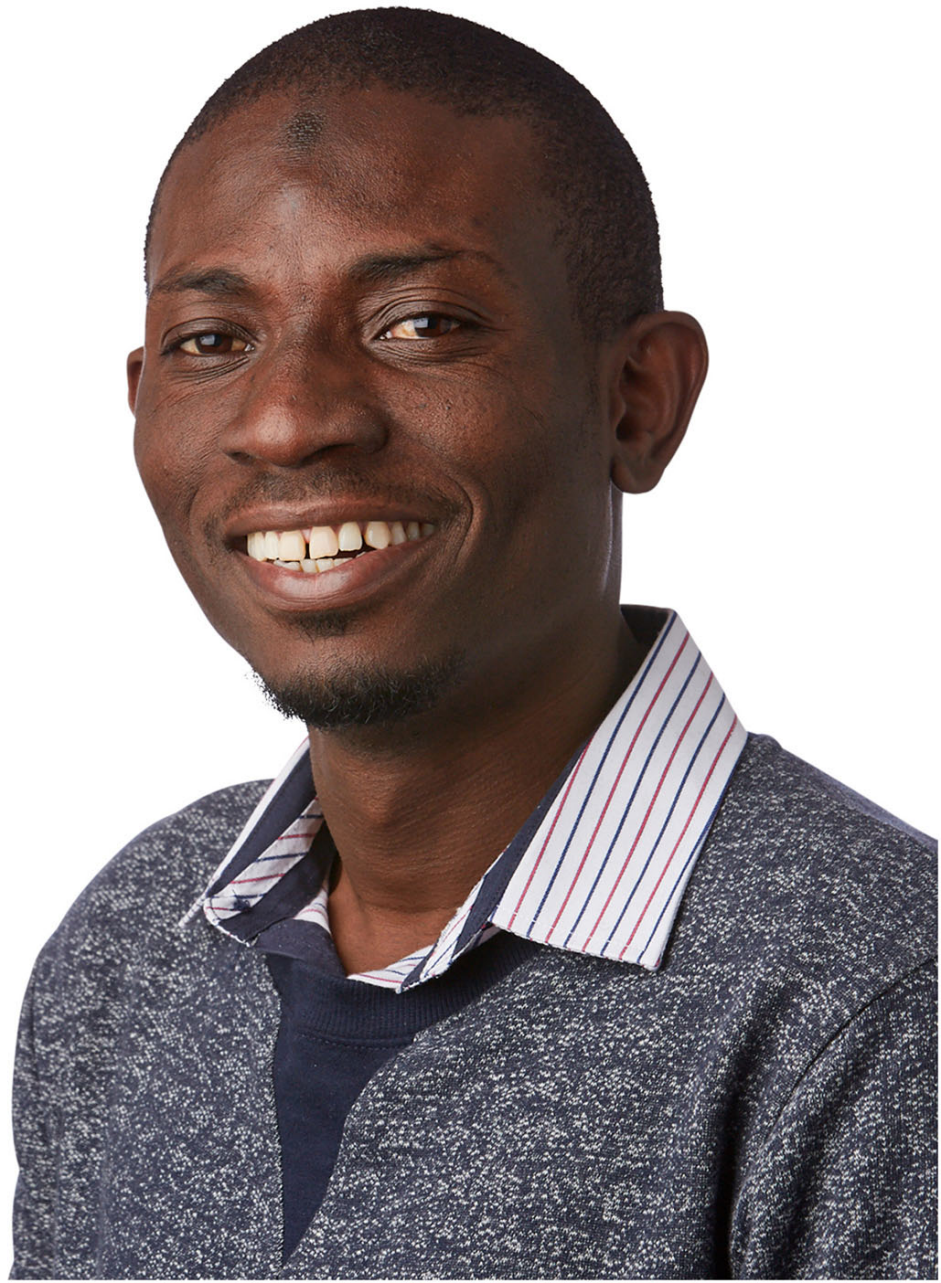


**Abdulsalam Isiaku**

**How would you explain the main findings of your paper to non-scientific family and friends?**

Zebrafish are a valuable model for studying white blood cells, mainly because their highly conserved genes can be edited to model human blood diseases. One major challenge in understanding the role of white blood cells in inflammatory and infective diseases has been the lack of well-characterised, flexible systems to accurately and concurrently edit genes in specific white blood cell types, rather than in the whole organism. We successfully generated tools based on a CRISPR-Cas9 technology for prompt and flexible gene editing in zebrafish white blood cells, particularly neutrophils and macrophages. These tools will greatly improve understanding of the genetic requirement of individual neutrophil and macrophage functions during development and disease.

“The new zebrafish tools are potentially useful in discovering new genes with cell-specific function in neutrophils and macrophages.”

**What are the potential implications of these results for your field of research?**

The new zebrafish tools are potentially useful in discovering new genes with cell-specific function in neutrophils and macrophages. Novel cell-intrinsic expressed genes may be important as markers during infectious and inflammatory disease processes, in monitoring and evaluating therapeutic interventions and/or guiding prognosis. Our system efficiently works using some synthetic components, speeding up the experimental pathway. This improvement for promptly disrupting gene function in zebrafish neutrophils and macrophages provides an *in vivo* platform that could be used for more efficient screening of potential therapeutic targets, diagnostic and prognostic markers of disease. Genes with restricted but essential neutrophil or macrophage functions are attractive therapeutic targets for improving inflammatory responses to foreign antigens (bacteria, viruses, parasites, tumours, chemicals) or promoting healthy repair of damage tissues following injury.

**What are the main advantages and drawbacks of the model system you have used as it relates to the disease you are investigating?**

The main advantage of these zebrafish gene-editing tools over others is that they provide opportunity to more promptly investigate the cell-intrinsic function of candidate genes in neutrophils and macrophages. In addition, the system can be combined with any of the many other already existing transgenic Gal4 driver lines to achieve gene editing in tissues other than neutrophils and macrophages.

One disadvantage is that because the genetic drivers of gene editing are expressed in more mature white blood cells, these systems are not expected to be as useful for studying gene function of myeloid progenitors in early white blood cell development. In common with all approaches for testing the cell-specific requirements of a gene, these tools cannot tell whether a gene is sufficient for that particular function – for this, gene overexpression studies must be used to complement findings.

**What has surprised you the most while conducting your research?**

**Figure:**
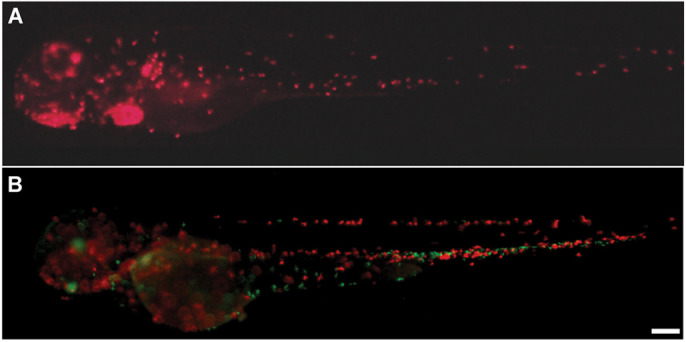
**Zebrafish embryos for gene editing in neutrophils (A; red dots, Cas9 marked by red heart) and macrophages (B; red dots, Cas9 marked by green eye, green dots are neutrophils).** Scale bar: 200 µm.

First, it was a useful surprise that microinjected synthetic guide RNA delivery in the leukocyte lineage-specific Cas9 transgenic embryos worked to achieve meaningful, functionally significant gene editing. More surprising was the fact that synthetic guide RNA was more effective than plasmid DNA delivery for gene editing, although we only tested this one way. I was also surprised that a similar level of gene editing could be achieved in both neutrophils and macrophages, despite the lower expression of gene-editing apparatus in macrophages compared to neutrophils.

**Describe what you think is the most significant challenge impacting your research at this time and how will this be addressed over the next 10 years?**

There are still gaps in the availability of zebrafish tools for studying neutrophils and macrophages. These white blood cells have several subtypes with specific functions depending on context, and the tools for separating these are not yet as well developed in zebrafish models and in mammalian systems. One reason for this is that antibodies for characterizing lineage subtypes, such as those used in mouse and human models, are unavailable in zebrafish. We need tools that can allow us to segregate each lineage subtype in a physiologically neutral setting. To some extent, advances in molecular technologies, including the promise of single-cell sequencing, could in the future help achieve significant progress in segregating and understanding the functions of genes in these lineage subtypes using zebrafish models.

“Creating a scientific environment that prioritises mental health will improve the lives of early-career researchers and retain them in the work force.”

**What changes do you think could improve the professional lives of early-career scientists?**

It is common knowledge that a lot of early-career researchers are leaving science. For some, this is associated with poor mental health. The mental stress is especially unbearable during the COVID-19 pandemic, particularly for those that are further away from their families. Creating a scientific environment that prioritises mental health will improve the lives of early-career researchers and retain them in the work force. Another area is in mentorship and funding of early-career scientists at both pre- and post-doctoral stages. Most early-career researchers are employed on insecure short-term contracts, and some bright minds are lost to more stable jobs outside academia/industry. Early-career scientists need improved job security and dedicated funding for them to successfully pursue their career goals.

**What's next for you?**

Science is key to solving many health challenges affecting the world today. This is most exemplified by the role of biomedical scientists in controlling the COVID-19 pandemic. I plan to continue in biomedical research, hopefully using the zebrafish to understand complex physiological processes and the role of white blood cells in disease. I qualified as a veterinarian before going into biomedical research, so this may be complemented by locum veterinary clinical practice.
